# Candidaemia in a tertiary hospital in Nigeria

**DOI:** 10.4102/ajlm.v3i1.89

**Published:** 2014-08-06

**Authors:** Rita O. Oladele, Rashidi A. Bakare, Michael A. Petrou, Oyinlola O. Oduyebo, Malcolm Richardson

**Affiliations:** 1 Department of Medical Microbiology and Parasitology, University College Hospital, Nigeria; 2 Department of Microbiology, Hammersmith Hospital, Imperial College, London; 3 Department of Medical Microbiology, College of Medicine, University of Lagos, Nigeria; 4 Mycology Reference Laboratory, University of Manchester, United Kingdom

## Abstract

**Background:**

Candidaemia is a widely-studied and reviewed topic in the developed world; however, there is a dearth of information on nosocomial candidaemia in Nigeria, despite the increasing use of more invasive therapeutic modalities, immunosuppressive agents and increasing incidence of immunosuppression as a result of malignancies and HIV.

**Objectives:**

To determine the hospital-based frequency of candidaemia in a tertiary hospital in Ibadan, Nigeria.

**Method:**

This was a prospective descriptive study which included 230 immunosuppressed patients. All isolates were identified to the species level using both conventional and automated methods. Thereafter, all *Candida* species isolated were tested for antifungal susceptibility using the broth microdilution method.

**Results:**

Candidaemia occurred in 12 (5.21%) of the 230 study patients, with *C. tropicalis* accounting for 50% of the infections. Four patients (33.3%) presented with *C. parapsilosis*, one (8.3%) with *C. albicans* and one (8.3%) with a mixed infection of *C. albicans* and *C. tropicalis*. All 12 isolates were sensitive to fluconazole (minimal inhibitory concentration < 8 mg/mL). Univariate analysis revealed that old age, multiple surgeries and long-term hospitalisation were significant contributing factors for the occurrence of candidaemia. Eleven (91.7%) of the 12 patients with candidaemia had *Candida* colonisation of other sterile sites including the bladder, peritoneum and trachea. Furthermore, bivariate analysis revealed that mucositis (*p* = 0.019) and diarrhoea (*p* = 0.017) were significantly associated with an increased risk of candidaemia. The crude mortality rate of candidaemia was 91.7%.

**Conclusion:**

This study highlights the significance of nosocomial candidaemia and the need for proactive laboratory investigation and clinical management of this life-threatening disease.

## Introduction

Since the 1980s, *Candida* infections have become a growing problem.^1,2,3,4^ Martin et al. reported a 207% increase in incidence between 1979 and 2000 in the United States.^5^ Morbidity rates in immunocompromised patients are as high as 50%.^6,7^ Patients with underlying clinical conditions, such as haematological malignancies, long-term intensive care unit (ICU) stays and prematurity, are at high risk for candidaemia. Independent risk factors for infection include treatment with broad-spectrum antibacterial agents, immunosuppressive therapy and/or parenteral nutrition; prior haemodialysis; and having implanted prosthetic devices.^8,9,10,11,12^

Candidaemia poses a complex clinical puzzle. Firstly, blood culture, the gold standard for diagnosis and treatment of candidaemia, is positive in less than 50% of infected patients and takes several days for a diagnosis.^13^ Second, trends in incidence and species distribution differ between geographical zones and healthcare facilities.^14^ Thirdly, some species of *Candida* are resistant to fluconazole. Without rapid diagnosis and treatment, the consequences are clear: a 20% increase in mortality was found in cases where antifungal therapy was delayed for more than 12 hours.^15,16^

As there is insufficient data on nosocomial candidaemia in Nigeria, we conducted a prospective study to establish the frequency of candidaemia, predisposing factors and the susceptibility of *Candida* spp. to fluconazole.

## Research method and design

Conducted from 01 September 2008 to 30 August 2009 at University College Hospital, a 960-bed, tertiary referral centre in Ibadan, Nigeria, this study enrolled 230 immunosuppressed patients who had been admitted at least 10 days prior to the start of the study and had persistent pyrexia (temperature > 38 °C). Study subjects included patients presenting with haematological malignancies; ICU patients; very low birth weight (VLBW) premature neonates (temperature > 38 °C or < 35 °C); patients who had undergone multiple abdominal surgical procedures during their current admission; diabetic patients with persistent pyrexia; and HIV patients. The patients’ underlying diseases were classified broadly, as can be seen in Table 1. Exclusion criteria included non-consenting patients, patients without clinical signs of infection and patients already receiving empirical antifungal therapy. Probability proportionate to size was used to estimate the number of patients selected per targeted study population group.^17^

**TABLE 1:** Demographic characteristics of patients studied and underlying diseases.

Demographic characteristics	Frequency	Percentage (%)
**Age in years**		
< 1	63	27.5
1–5	23	10.0
6–20	31	13.5
21–40	42	18.3
41–60	45	19.7
> 60	25	10.9
**Sex**		
Male	125	55.3
Female	101	44.7
**Number of days of admission**		
0–10	64	28.3
11–20	89	39.7
21–30	35	14.2
30 +	42	17.8
**Underlying disease**		
Systemic infection	50	21.7
Sepsis	35	15.2
Solid tumours	30	13.0
Haematological malignancy	25	10.9
Very low birth weight	25	10.9
HIV	24	10.4
Renal diseases	19	8.3
Diabetes mellitus	12	5.2
Severe burns	5	2.2
Severe head injury	5	2.2

Participation in the study was voluntary and, after receiving written consent from the patients or their immediate relatives, the investigators explained the study in both English and Yoruba and then administered a semi-structured questionnaire, adapted from a European Confederation of Medical Mycology Intensive Care Unit (ECMM ICU) form. The questionnaire was divided into three sections: *Socio-demographic Data*; *Medical and Surgical History* (based on known risk factors for candidaemia); and *Laboratory Findings*.

Candidaemia was defined as being the isolation of *Candida* species from at least one positive blood culture sample in patients with clinical signs of bloodstream infection. Two venous blood samples of five mL each were collected from each patient. Ten millilitres of blood was added to each BACTEC blood culture bottle and incubated at 37 °C using a BACTEC 9050 blood culture system (Becton Dickinson, Inc., Sparks, MD, USA), an automated system that contains antibiotic-absolving resins, allowing for the culturing of blood from patients on antibiotics without discontinuing treatment. Additional advantages of this system are its rapid turnaround time and increased yield compared with the conventional, manual blood culture system. Positive samples were examined microscopically using direct gram staining; those showing yeast were cultured onto Sabouraud’s dextrose agar (Oxoid, UK) and CHROMagar *Candida* (CHROMagar, France). The germ tube test was used for presumptive diagnosis of *C. albicans*, but all isolates were identified to the species level using API 20C AUX and/or ID 32C automated strip detection (BioMerieux Vitek, Inc., St Louis, MO, USA).

Susceptibility testing to fluconazole was performed using the broth microdilution method in accordance with the Clinical and Laboratory Standards Institute (CLSI) recommendations (M27-A2).^18^ Stock inocula were prepared by adding 24–48-hour-old test *Candida* to Roswell Park Memorial Institute (RPMI) broth and adjusting to a 0.5 McFarland standard. The stock inocula were diluted 1:1000 and 100 μL of each was added to two-fold 100 μL dilutions (ranging from 0.12 to 64 μg/mL) of fluconazole on the microtitre plate as well as to a drug-free medium. After incubating the microtitre plates at 35 °C for 24–48 hours, the amount of growth in a well containing the antifungal agent was compared with the amount of growth in an antifungal-free, growth-control well. The minimum inhibitory concentration (MIC) was the lowest concentration of antifungal agent that visibly inhibited 50% growth of the organism. Isolates with an MIC < 8 mg/mL were considered to be susceptible to fluconazole; isolates with an MIC > 64 mg/mL were resistant.^18^ Those with an MIC from 16–32 mg/mL were fluconazole susceptible dose-dependent (S-DD).^18^ Quality control was ensured by the inclusion of CLSI-recommended quality control strains *Candida parapsilopsis* ATCC 22019 (MIC range 2–8 mg/mL) and *Candida krusei* ATCC 6258 (MIC range 16–64 mg/mL).

Quantitative data from the questionnaire were entered into Microsoft Excel 2003 (v11.0) (Microsoft, Redmond, WA) and SPSS 15.0 for Windows (SPSS Inc., Chicago, 2006) was used for the analysis. The univariate analysis used descriptive statistics and tables, including frequency distribution; and the bivariate analysis used the chi-square test with statistical significance set at *p* < 0.05.

## Results

Of the 230 study patients, the median age was 18 years, with 27.5% (*n* = 63) being less than one year old and 10.9% (*n* = 25) older than 60. The male-to-female ratio was 1.12:1. All patients were admitted for hospitalisation: 39.7% (*n* = 89) for 11–20 days and 17.8% (*n* = 42) for more than 30 days (Table 1).

Seven percent (*n* = 16) of the patients underwent more than one operation (Table 2). Most of the study population (97.4%; *n* = 224) were treated with broad-spectrum antibiotics; 45.7% (*n* = 105) of these received 11–20 days of different combination of broad-spectrum antibiotics. The median duration of antibiotic use was 15.5 days, with only 11.7% (*n* = 27) of patients receiving antibiotic treatment for more than 21 days (Table 2). Most patients had at least one invasive procedure during their admission, with 92.2% (*n* = 212) undergoing intravenous cannulation and 8.7% (*n* = 7) undergoing venous cutdown (Table 2). Other treatments used in this population included antifungal therapy for non-invasive candidiasis, anti-cancer chemotherapy, radiotherapy, steroids and highly-active antiretroviral therapy (HAART).

**TABLE 2:** Treatment history of patients in the study.

Clinical Characteristics	Frequency	Percentage (%)
**Number of surgical operations**		
None	165	71.7
1	49	21.3
> 1	16	7.0
**Duration of broad spectrum antibiotic therapy**		
1–10 days	92	42.6
11–20 days	105	45.7
21–40 days	27	11.7
**Invasive procedures**		
Intravenous cannulation	212	92.2
Nasogastric tube passage	143	62.2
Urinary catheterisation	118	51.8
Tracheostomy	20	13.6
Venous cutdown	7	8.7

**FIGURE 1:**
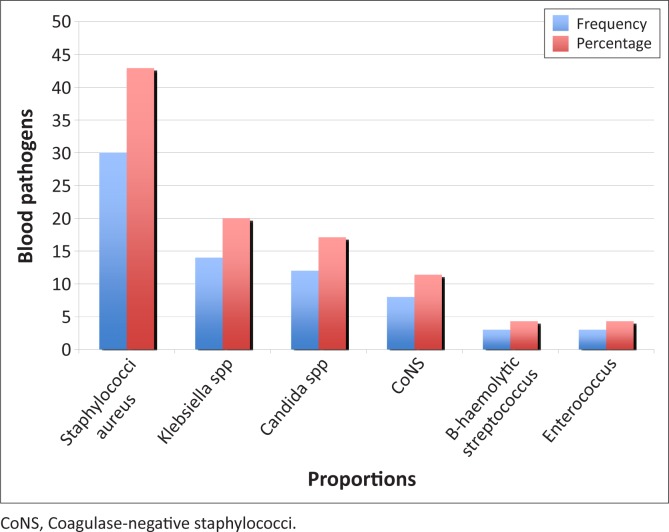
Bar chart showing distribution of pathogens causing bloodstream infections.

**FIGURE 2:**
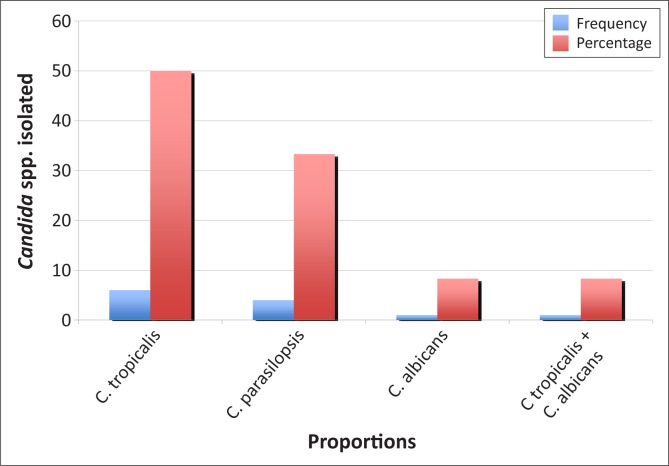
Bar chart showing distribution of *Candida* spp. causing bloodstream infections.

**FIGURE 3:**
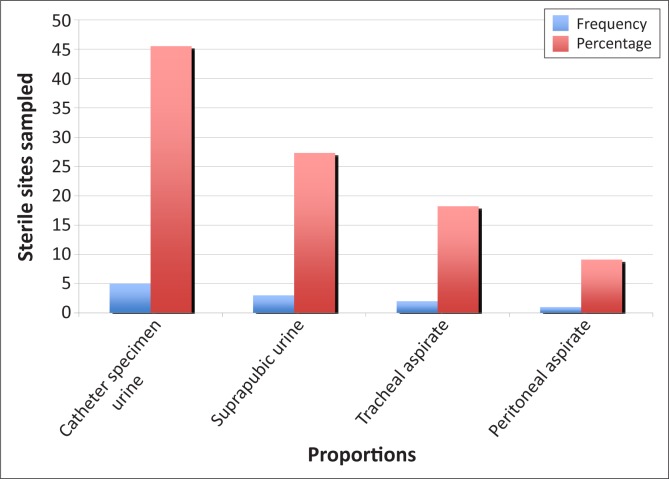
Bar chart showing distribution of *Candida* spp. isolated from other sterile sites.

The distribution pattern of isolates from blood culture samples showed that *Staphylococcus aureus* (42.9%; *n* = 30) and *Klebsiella* spp. (20.0%; *n* = 14) were the most common pathogens, followed by *Candida* spp. (17.1%; *n* = 12) (Figure 1). Of the enrolled patients, 12 (5.2%) had candidaemia. *C. tropicalis* (*n* = 6; 50%) was the most frequently-identified *Candida* spp. (Figure 2). Based on CHROMagar *Candida* phenotypes, one patient had a polymicrobial infection with both *C. albicans* and *C. tropicalis*. *Candida* spp. was also isolated from non-venous, invasive sites in 11 (91.7%) of the 12 patients (Figure 3). All *Candida* spp. isolates were susceptible to fluconazole (MIC < 8 mg/mL). In addition, the API 20C AUX and ID 32C identification of the *Candida* spp. were in concordance with one another.

Of the 12 (5.2%) patients with candidaemia in this study, age (*p* = 0.8) and sex (*p* = 0.16) distributions were not of statistical significance. These patients’ underlying diseases included solid tumours, diabetes, HIV, infective endocarditis, haematological malignancy, VLBW, severe head injury and sepsis. There was no significant relationship (*p* = 0.18) between underlying disease and the various *Candida* spp. detected. Using chi-square analysis, diarrhoea (*p* = 0.017) and oral thrush (mucositis) (*p* = 0.019) were the only statistically-significant symptoms (Table 3). The crude mortality rate was 91.7% (11 out of 12 patients).

**TABLE 3:** Association of symptoms of immunosuppression in patients who developed candidaemia.

Signs	Symptoms	*Candida* positive		*Candida* negative		Chi-square value	*p*-value
		*n*	%	*n*	%		
Diarrhoea	Yes	4	14.8	23	14.8	5.698	0.017
	No	8	3.9	195	3.9		
Fever	Yes	9	4.6	186	95.4	0.936	0.333
	No	3	8.6	32	91.4		
Lethargy	Yes	8	6.6	113	93.4	1.004	0.316
	No	4	3.7	105	96.3		
Weight loss	Yes	7	5.4	123	94.6	0.017	0.897
	No	5	5.0	95	95.0		
Loss of appetite	Yes	4	4.9	78	95.1	0.030	0.863
	No	8	5.4	140	94.6		
Skin rashes	Yes	0	0.0	19	100.0	1.140	0.286
	No	12	5.7	199	94.3		
Boils/ carbuncles	Yes	0	0.0	7	100.0	0.397	0.528
	No	12	5.4	211	94.6		
Oral thrush	Yes	5	12.8	34	87.2	5.490	0.019
	No	7	3.7	184	96.3		
Sore throat	Yes	0	0.0	5	100.0	0.281	0.596
	No	12	5.3	213	94.7		
Cough	Yes	2	6.9	27	93.1	0.189	0.664
	No	10	5.0	191	95.0		
Hypothermia	Yes	1	2.6	34	97.4	0.669	0.414
	No	11	5.8	180	94.2		
Feed intolerance	Yes	0	0.0	2	100.0	0.111	0.739
	No	12	5.3	216	94.7		
Loss of consciousness	Yes	5	7.8	59	92.2	1.208	0.272
	No	7	4.2	159	95.8		
Vomiting	Yes	1	4.2	23	95.8	0.060	0.807
	No	11	5.3	195	94.7		

## Ethical considerations

Ethical approval for the study was obtained from the National Health Research Ethics Committee (NHREC/ 05/01/2008a) and, in keeping with ethical procedures, the patients or their immediate relatives provided informed written consent.

### Potential benefits and hazards

The sample collection is a minimum risk procedure (venipuncture under standard precautions). Benefits to the participants included free blood culture and feedback of results to the managing clinicians.

## Trustworthiness

Confidentiality was ensured by the identification of participants with code numbers. Other personal details of the participants were kept in safe keeping with the lead researcher for 5 years in a secured file.

## Discussion

Candidaemia is a nosocomial infection commonly seen in critically-ill patients and those with haematological malignancies.^1,2,4,6,19^ In this study, *Candida* spp. ranked third among pathogens causing blood stream infections (BSIs) in hospitalised patients admitted to the study centre. Findings from North American and European surveillance programmes of hospital-acquired infections showed that isolates of *Candida* spp. were the fourth most common cause, accounting for 8% – 10% of nosocomial BSIs.^19^ This difference could exist because all of the participants in this study were immunosuppressed; not all presented with BSIs; and the developed world has established protocols and guidelines for the diagnosis, management and prevention of invasive fungal infections.

The hospital-based frequency of candidaemia in this study was 5.2%, which could possibly be a result of Nigeria’s progressive changes in the medical and surgical management of patients over the last five years. These improvements have reduced the vulnerability of critically-ill patients to haematogenous dissemination of *Candida* spp. whereas previously, bacterial and/or viral infections often resulted in fatalities. Conversely, antifungal drugs are not administered routinely, either empirically or as prophylaxis, in the management of high-risk patients in Nigeria, which could possibly explain the high mortality rates from candidaemia.

Several risk factors for candidaemia have been shown in previous studies and confirmed in this study. In this study, patients under the age of one (*n* = 63) accounted for 27.5% of the study population and 25% of patients with candidaemia. This is consistent with findings by Saiman et al. which showed that infants, especially premature, VLBW babies, have a higher risk of candidaemia because of their immature immune systems and frequent intubation.^20^ Patients over the age of 60 (*n* = 25) accounted for 10.9% of the studied population and 16.7% of the cases of candidaemia, which is relatively high, but not of statistical significance; however, Schelenz found that old age was an independent risk factor for candidaemia.^21^

Two of the patients had infective endocarditis, one of whom had undergone cardiac surgery, a high-risk factor for fungal endocarditis, with an infection rate of 0.23% – 1.0%.^22,23^ Another known risk factor for candidaemia is long-term hospital stays, especially in ICU settings.^22^ There was a correlation between length of hospital stay and frequency of candidaemia, further confirming the nosocomial source of the infection and that prolonged hospital stays are risk factors.^2,3,19^

Broad-spectrum antibiotic use is a major risk factor for the development of candidaemia because these medications eliminate the bacterial gut flora and encourage the overgrowth and dissemination of gastrointestinal fungal commensals.^19,21^ Almost all of the enrolled patients with candidaemia were taking several antibiotics, for a median period of 15.5 days.

Most patients had intravenous lines *in situ*, but only 13.6% (*n* = 7) underwent intravenous cutdown (femoral vein). 11 of the 12 patients with candidaemia had the same *Candida* spp. isolated from other sites. Symptoms associated with candidaemia were mucositis (*p* = 0.019) and diarrhoea (*p* = 0.017). These findings are consistent with those of other reports showing mucosal colonisation to be an independent risk factor for candidaemia;^21,24^ however, there are no previous studies citing any relationship between candidaemia and diarrhoea.

The high proportion of *C. tropicalis* (50%) reported here is consistent with a study in India by Verma et al. where the proportion was 46%.^25^ The crude mortality rate in our study was 91.7% (11 out of 12 patients) compared with the 35% – 75% crude mortality rates reported in other studies.^23^ This could be attributed to small sample sizes, as well as to the fact that the clinicians at the study site did not administer antifungal drugs routinely, either on an empirical or a prophylactic basis, for the management or prevention of candidaemia. Clinicians may not consider candidaemia as a cause of infection because of limited training in nosocomial diseases, the absence of knowledge of disease prevalence (NCBI Pubmed searches reveal few publications in the field from Nigeria) and the lack of an established infrastructure for high-quality laboratory testing.

## Limitations of the study

Limitations of this study include the small sample size and generalised study population.

## Conclusion

This study highlights the clinical significance of nosocomial candidaemia and the need for proactive investigation for possible invasive candidiasis. This is particularly needed in Nigeria where patients have to pay for every laboratory investigation carried out in the hospital; as a result, clinicians cannot proactively and/or repeatedly request tests or cultures. This study highlights the need for affordable laboratory tests and an improved understanding of the dangers of untreated candidaemia. Finally, improved communication and collaboration between clinicians and clinical microbiologists is essential for the development of measures to control the emerging threat of nosocomial candidaemia.
